# Health, Climate Change and Sustainability: A systematic Review and Thematic Analysis of the Literature

**DOI:** 10.4137/EHI.S3003

**Published:** 2009-08-24

**Authors:** A. Nichols, V. Maynard, B. Goodman, J. Richardson

**Affiliations:** 1 Faculty of Health and Social Work, University of Plymouth, 3 Portland Villas, Drake Circus, Plymouth, Devon PL4 8AA, U.K; 2 School of Health Professions, University of Plymouth, 19 Portland Villas, Drake Circus, Plymouth, Devon PL4 8AA, U.K; 3 School of Nursing and Community Studies, University of Plymouth, FHSW L4, Knowledge Spa, RCHT Treliske, Truro, Cornwall TR1 3HD, U.K; 4 Professor of Health Services Research, Faculty of Health and Social Work, University of Plymouth, 19 Portland Villas, Drake Circus, Plymouth, Devon PL4 8AA, U.K

**Keywords:** sustainability, climate change, health, systematic review

## Abstract

**Background:**

Evidence of climate change and its impact continues to be accumulated, and it is argued that the consequences of climate change are likely to result in an increased demand on health services. It has been claimed that climate change presents new challenges for health services and that strategies should be adopted to address these challenges.

**Aim:**

The aim of this systematic review was to map published literature on health, climate change and sustainability by categorising papers according to their focus on effects, strategy and actions, and to provide a thematic analysis of their content.

**Methods:**

Systematic searches were conducted via a range of healthcare related databases i.e. Pubmed, Medline, CINAHL, AMED, ASSIA, IBSS and ISI Web of Knowledge. Searches focussed upon papers published in English between 1998 and 2008. Retrieved papers were studied by the authors in order to inform the thematic analysis of their content.

**Results:**

A total of 114 publications were retrieved, of which 36 met the inclusion criteria for the systematic review. These 36 publications were categorised and are discussed according to their focus on: effects/impacts, strategy/policy, action/examples.

**Conclusions:**

A number of papers report the potential health effects of climate change while others report policies and strategies to tackle these effects. However there is an urgent need to identify and report on the implementation of strategies to mitigate and adapt to these challenges and to publish real examples of actions. Actions that are taken need to be evidence/policy based, and implementations monitored, evaluated and published.

## Introduction

As evidence of climate change and its impact continues to be amassed it has become clear that many of the causes of climate change are anthropogenic in nature through lifestyles, consumption and choices that pollute and exploit resources in an unsustainable manner.^1^–^3^ The future health impacts of climate change are well documented, with forecasts made of increasing health problems caused by heatwaves, storms, floods, fires, droughts and infectious diseases.^4^–^6^ It is also predicted that climate change will have detrimental effects upon agriculture and fisheries, and may even result in collapsing ecosystems.^4^,^5^ In terms of economics, Roberts^7^ argues that while the costs of tackling climate change may be great, they will be much less than the costs incurred through inaction, and as Stott and Godlee^8^ claim, could be as much as 20% of the global gross domestic product. The effects of climate change are not limited solely to the realms of economic or potential future impacts. Some consequences and health impacts of climate change have already been observed. For example, in episodes of flash flooding and outbreaks of *Escherichia coli* 0157 in the United Kingdom (UK) and in the increasing incidence of tick borne infections throughout Europe.^3^,^9^

The consequences of climate change are likely to lead to an increased demand on emergency and health services. Add to this the challenges of meeting local healthcare needs in a post peak-oil scenario, with possible limited access to medicines, transport and energy difficulties, and we could be facing a massive public health disaster which will need to be addressed by Health Services.^5^,^10^,^11^

The UK National Health Service (NHS) however continues to make its own contribution to the problem of climate change with recent estimates suggesting that the NHS is responsible for 5% of the road transport emissions in the UK and that in total it emits around a million tonnes of carbon each year.^11^,^12^ In addition to this, it is claimed that the NHS annually spends up to £400 million on energy costs.^11^,^12^ It has been asserted that climate change is a “new front for health” p205^3^ and that strategies should be adopted with a view to addressing the challenges and consequences of unsustainable consumption and climate change.^1^,^13^ These strategies should include measures to mitigate and adapt to climate change, and it has been claimed that with its large estate, purchasing power and significant numbers of employees (more than 1.3 m people) the UK NHS has enormous power to mitigate the impact of climate change by implementing sustainable practices and encouraging its employees to do likewise.^14^ A potentially significant contribution to focused NHS action on climate change is the establishment of the NHS Sustainable Development Unit (http://www.sdu.nhs.uk/) and the recent launch of the NHS Carbon Reduction Strategy for England (http://www.sdu.nhs.uk/page.php?page_id=94).

The responsibility of healthcare practitioners to protect and promote the health of the public should be extended to working to prevent climate change according to some authors.^15^ In pursuit of this aim, the UK Public Health Association^16^ outlined strategies for promoting health and sustainable development, and the Climate and Health Council has been established as a not-for-profit international organisation aiming to mobilise health professionals across the world to take action to limit climate change and its effects on human health (http://www.climateandhealth.org/). The UK Department of Health has produced a Guidance Document on The Health Impact of Climate Change: Promoting Sustainable Communities^17^ and the British Medical Journal (BMJ) has set up a carbon council aimed at exploiting the knowledge and creativity of healthcare workers in achieving a transition to a more sustainable world.^18^ In addition, efforts are being made to reduce the carbon footprint of attending medical conferences.^19^

Instances of good practice in addressing sustainability and climate change are found within the UK NHS. For example, Cornwall PCT has taken action to provide sustainable waste management and locally sourced food^20^ (http://www.cornwallandislesofscilly.nhs.uk/RoyalCornwallHospitalsTrust/OurOrganisation/NewsAndPublications/Publications/AnnualReports/AnnualReport0708/FitterFuture/BuildingASusstainableFuture/FoodUnitLeadsTheWayForNHS.aspx), and the Gwent Healthcare NHS Trust 25-year performance contract with energy services company, Honeywell, has enabled the introduction of dual fuel burners, a combined heat and power plant and seen the rollout of water conservation measures across the organisation (http://www.healthexec.tv/cgi-bin/details.pl?action=pre&id=426). However, such instances remain comparatively rare and it has been argued that organisations responsible for commissioning and providing healthcare services in the UK such as Strategic Health Authorities and Primary Care Trusts have not yet fully considered the impacts of climate change or developed strategies aimed at promoting resilient and sustainable communities.^21^ This may be due in part to a reluctance amongst senior healthcare managers and clinicians to acknowledge that climate change will have unavoidable impacts upon healthcare in the UK, and/or a critical view taken by some of the nature and quality of evidence in relation to climate change and sustainability.^22^

The aim of this systematic review was to map published literature on health and sustainability by categorising papers according to their focus on effects, strategy and actions, and to provide a thematic analysis of their content.

## Methods

Systematic searches were conducted via a range of healthcare related databases and citations were sought from relevant reviews. During October and November 2008 the following databases were searched—Pubmed, Medline, CINAHL, AMED, ASSIA, IBSS and ISI Web of Knowledge. These searches focussed upon papers published between 1998 and 2008.

### 

#### Search terms

Health, healthcare, health care, health services, sustainability, climate change, peak oil, energy vulnerability, mitigation, adaptation.

#### Inclusion criteria

- Papers reporting the global impacts of climate change/sustainability on physical, mental and public health
- Papers reporting the global impacts of climate change/sustainability upon the health environment
- Papers based on analysis and discussion of climate change/sustainability in relation to health and health care (practice, leadership, education, research in the UK
- Papers focussing on climate change/sustainability that report on outcomes/measures that may include: interventions or developments, patient or staff outcomes or views, contributions to research, demonstrations of service developments, development of educational interventions/packages; policies/strategies and their implementation in the UK
- Papers that include a cost-benefit analysis/health economic appraisal of climate change/sustainability in relation to health care in the UK
- Papers published in English
- Papers published since 1998

#### Exclusion criteria

- Papers reporting strategies and actions that were not UK-based
- Editorials and letters
- News articles
- Non English language papers
- Papers published before 1998

#### Filtering

Citations were filtered independently by two members of the research team (JR and VM). Where there were discrepancies, the full article was retrieved.

### Data collection and analysis

Data was extracted systematically using a specially designed data extraction form and categorised according to the focus of the publication: Effects; Strategies/Policies; Actions. Following this categorisation a thematic analysis was conducted in order to identify key issues, policies and actions. For each publication, data extraction and thematic analysis were conducted independently by two researchers (JR, VM) and any disagreements or discrepancies were resolved by discussion and involvement of the third researcher (AN). The thematic analysis^23^ was undertaken in accordance with methods used previously.^21^ Themes emerging from the publications were extracted, together with a summary to illustrate each theme. Due to the heterogeneity of the papers and the absence of evaluation studies a quality assessment of the included papers was not undertaken.

## Findings

**Figure 1.**
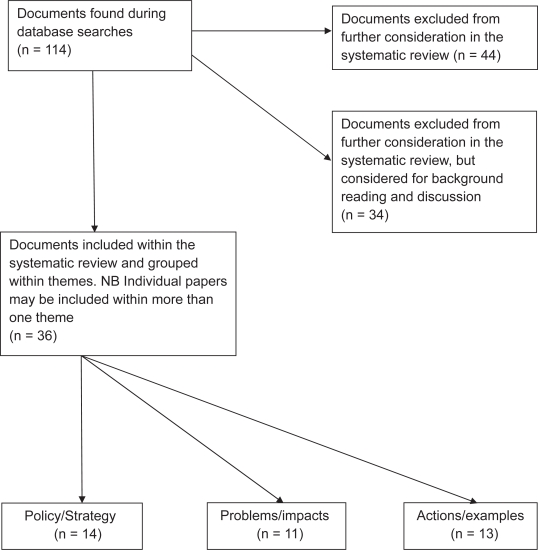
Systematic review search results flow chart.

A total of 114 studies were retrieved for possible inclusion, of which 36 actually met the inclusion criteria (see Fig. 1).

Many of the retrieved papers were comments or editorials and were therefore excluded (see Table 1). The 36 publications were categorised according to their focus on: effects/impacts, strategy/policy, action/examples (Table 2). This categorisation was consistent with previous work examining the action being taken by Strategic Health Authorities and Primary Care Trusts.^21^,^24^

### Global effects/impacts

A number of publications attempted to provide details of the potential effects of climate change and energy vulnerability on health, with the main health effects being grouped under the following themes: waterborne disease; vector borne disease; food borne disease; incidence of temperature related deaths; skin cancer and cataracts; increase in starvation and malnutrition; increased incidence of respiratory disorders/death due to pollution/particulate matter; injury and death due to flooding/storms; psychological effects. Table 3 provides a summary of examples of the global impacts and effects of climate change in relation to health as reported in the literature reviewed.

#### Waterborne disease

The increase in ambient temperature and flooding associated with climate change is likely to result in an increase in the spread of vectors such as mosquitoes, with a resultant increase in incidence of water-borne diseases such as malaria,^2^,^25^,^26^ particularly in developing countries;^27^,^28^ it is unlikely however, that in those countries that have adequate public health and medical infrastructures there would be a significant spread of malaria.^27^ Nevertheless, Campbell^25^ highlights the need for vigilance throughout Europe with the possibility of the arrival of new species of mosquito that could act as more effective vectors. Similarly, the increased risk of infection due to flooding is most likely to affect poorer countries and not the UK or the West unless water sources themselves are breached.^27^ The effect of rising temperatures on the development of toxic blooms that are detrimental to health have also been highlighted by Hunter,^27^ along with an increased incidence of cholera associated with such blooms.

#### Vector borne disease

Campbell^25^ reports that changes in human behaviour associated with alterations in land usage and leisure activity due to warmer temperatures in the UK are likely to have the greatest impact on the transmission of vector-borne diseases such as that spread by insects (e.g. flies, ticks and mosquitoes).^25^ In addition, there have been reports of vector species responding to climate change in Europe, with latitudinal shifts in the population of ticks due to rising temperatures, resulting in changes in the incidence, distribution and transmission of tick-borne encephalitis.^26^ However, the likelihood that tick-borne encephalitis would become a major problem in the UK is probably very limited.^26^ Factors such as temperature, humidity, levels of precipitation, soil moisture and sea level rise can all have an impact on the transmission of vector-borne infectious diseases, so determining how these factors may affect the risk to populations is not straightforward. Added to these environmental factors, there are also a number of plausible alternative explanations for changes to vector species, such as changes to land usage and socio-economic and demographic factors as outlined by Haines et al.^29^

#### Food borne disease

A number of authors report an increase in death and disease from food poisoning associated with climate change and the effects of flooding^25^,^30^ as well as the effects of higher temperatures on food storage and food hygiene.^2^ Rising temperatures and increased risk of flooding are also likely to affect the distribution and incidence of diarrhoeal disease, with associated increased risk of food contamination.^2^,^26^–^28^ It is expected that the greatest risk of diarrhoea will occur in populations in developing countries, with Western countries suffering little or no additional risk.^26^ Food contamination may also result from flooding with the remobilisation of chemicals and pesticides on the land, particularly in areas where industrial or agricultural land directly abuts residential land. However there has been little research in this area to determine the causal effect of chemical contamination on health of neighbouring communities.^26^

#### Incidence of temperature related deaths

Death due to increases in temperature and humidity associated with climate change and more frequent heat waves are predicted with many of the actual causes of death being due to cardiovascular, cerebrovascular and respiratory events which will predominately affect the most vulnerable populations such as the elderly.^26^,^28^,^29^ Urban areas are more likely to be affected than rural or sub-urban areas due to the ‘heat island effect’ and the increase in air pollution resulting in respiratory deaths.^29^

There is some suggestion that warmer temperatures associated with climate change will bring about a reduction in cold-related deaths, particularly in the elderly (e.g.^26^,^28^,^31^ though it is expected that these benefits are likely to be very small.

#### Skin cancer and cataracts

It is reported that modifications to health behaviours as a result of rising temperatures associated with climate change will lead to an increase in human UV exposure as individuals spend more time in the sun. This increased exposure to UV light, along with the ozone depletion also associated with climate change, is likely to result in an increase in incidence of skin cancer and cataracts.^32^,^33^

#### Increase in malnutrition/starvation

The effect of climate change and weather extremes on agricultural productivity will have a direct impact on the health and nutrition of the population.^25^,^26^ Such effects will have greatest impact on agriculture and crop yields in areas such as Africa and other developing countries already affected by drought and conflict.^25^ Droughts will have wide ranging effects on health including nutrition and famine^26^ with crop failures having worldwide impact resulting in food insecurity and food shortages.^25^ Rising sea levels and flooding in coastal areas may impact on local agricultural infrastructure^25^ and will affect local biodiversity and ecosystems with a knock on effect on goods and services.^26^ Reduction in yields from agriculture result in reduced availability of food and increase in malnutrition and consequent disorders with implications for child growth and development.^28^ There is also likely to be an increase in armed conflict over water, land and food supplies, resulting in population migration and displacement.^25^

#### Increase in respiratory disease and deaths due to pollution/particulate matter

A predicted increase in cardio-respiratory and respiratory diseases is expected due to an increase in ground level ozone,^28^ an increase in general air pollution caused by more frequent forest fires,^26^ and an increase in particulate matter as a result of rising use of diesel fuel in the UK.^26^ However, one health benefit associated with rising temperatures that has been identified is the shortening of the respiratory syncytial virus (RSV) season.^34^

#### Injury/death due to flooding/storms

The direct and immediate effects of flooding and/or storms on health and well-being have been well documented, and include an increased risk of drowning and other injuries as well as potentially longer lasting effects on mental health.^26^ Contamination due to mobilisation of dangerous chemicals and increased risk of diarrhoeal and respiratory diseases have also been identified as a direct result of flooding.^26^

#### Psychological effects

The impact of flooding and the stress of population displacement on the psychological health of individuals is also well documented.^26^,^30^ For example, due to climate change and more frequent natural disasters, communities are displaced as result of flooding, drought and environmental degradation and this creates pressures in neighbouring areas as they struggle for survival or compete for employment. This population displacement and the creation of environmental refugees creates psychological and mental health problems for those concerned.^25^,^26^

### Strategy/policy

The majority of publications we found emphasised the need for policies and strategies; providing details of what these should include, or referencing specific UK Government policies (Table 4).

#### Food and health

Fairchild and Morgan^35^ describe a comprehensive food policy developed for Cardiff that encompasses sustainability, education, training, nutrition, food provision and food supply. The policy objectives included rapid appraisal of food initiatives, establishment of a food health strategy working group, and a preparation of food and health strategy. Their approach reinforces recommendations that policies should be guided by health and broader ecological sustainability.^35^ The impacts of poor food policy decisions on public health are outlined by Caraher and Coveney.^36^ They argue for the need for food policy to take into account the wider elements of the food system, such as the control of food supply, and the effects of globalisation on health. Haines et al^29^ consider the implications of climate change on food production, processing and distribution and outline the need for policies that increase food crop production and prioritise food security. With the possibility of some food crops being diverted to produce biofuels for transportation, there is a need to ensure food security in those areas and to support sustainable land-use policies that maintain and enhance food crop production.

#### Contraction and convergence

Contraction and convergence has been proposed as the most practical and equitable strategy for reducing CO_2_ emissions and tackling climate change.^18^,^37^–^40^ This approach has also been proposed by the Global Common Institute^37^ and would require the establishment of a global carbon budget with allocation of entitlement of carbon to each region, country or individual^18^ and major changes in all sections of the economy including: electricity production, transport, housing, agriculture, industry and commerce.^41^ Stott^18^ outlines the injury control benefits that would result from carbon rationing.

#### Waste and water management

Brayford^42^ examines sustainability in the UK NHS and considers actions and co-benefits around the five key themes of energy, water, waste, transport and procurement. In relation to waste management in the NHS, Brayford^42^ outlines the requirements placed upon NHS Estates who have been tasked with producing a strategy for ‘*Total Healthcare Waste Management*’ in order to enhance sustainability, to reduce waste disposal costs and reduce the environmental impact of the NHS whilst at the same time bringing about benefits to patient care and health services. There is also reference to further plans that have been proposed, such as *Waste Not, Want Not—A Strategy for Tackling Waste Production in England*^43^ that have been developed to reduce dependence on landfill. Mohan et al^44^ outlines the involvement of public health professionals in regulation of waste management as part of the Integrated Pollution Prevention and Control (IPPC) process which is designed to ensure that all waste management installations are operating so as to minimise risk to human health. Recommendations are made for the use of health impact assessments (HIA) to determine the impact that a waste management site could have on the health of a local community and for consideration of possible mitigation measures, such as the use of Life Cycle Assessments (LCA) to ensure a sustainable approach.^44^ The LCA approach takes into account the environmental impact of a product or service over its entire life cycle and provides an evidence based approach to waste management and planning for sustainability.

#### Sustainability

Brayford^42^ describes the latest strategy from NHS Estates: *New Environmental Strategy for the NHS* which encompasses buildings, energy, waste, water, transport and procurement and tasks the NHS with both reducing waste and enhancing efficiency in these areas, whilst at the same time bringing about patient benefit. A document produced to guide the NHS in its development of sustainability policies in these areas is *Sustainable Development in the NHS*.^42^ The need for sustainability policies covering such areas as energy, transport, town planning and structure of economy have been highlighted by Hanlon and McCartney^45^ as essential to address shortfalls in energy supply, rising energy prices and energy vulnerability associated with ‘peak oil’. Hanlon and McCartney^45^ emphasise that there is scope through economic planning and sustainable development to reduce the adverse effects of such changes on public health and that this should be tackled at both the national and international level through structures such as the European Union. Haines et al^29^ considers policies to promote access to clean and sustainable energy sources in order to improve public health and mitigate climate change. They report on a number of technology and policy options and economic instruments for the mitigation of greenhouse gas emissions and use of energy in different sectors, such as power generation, transport, agriculture, and the built environment, with policies to reduce human population growth and livestock production also potentially playing an important role in tackling climate change. Coote^46^ outlines the need for NHS policies that promote sustainable development to include all aspects of its business including: food for patients, cleaning equipment, pharmaceuticals, vehicles, childcare services, new buildings, energy, water and waste, and how it involves patients and public in decision making; and a sustainable health policy that gives priority to maintaining and improving health for all and reducing health inequalities.

#### Transport

Brayford^42^ outlines transport policies that are aimed at limiting environmental impact, reducing congestion and improving health. NHS philosophy is supported by the Government’s White Paper *New Deal for Transport: Better for Everyone* which emphasises the role that the NHS and its hospitals can play in encouraging a more sustainable approach to travel.^42^ The health benefits of transition to a low-carbon, low-energy transport system have also been highlighted by Roberts and Arnold.^47^ These benefits include a reduction in the volume and speed of traffic that could mitigate climate impacts, reduce injury rates and improved air quality whilst simultaneously improving health. Roberts and Arnold^47^ emphasise that increases in levels of active transport (such as walking and cycling) are key to limiting environmental pollution whilst at the same time bringing considerable health co-benefits. Co-benefits outlined include reductions in traffic pollution, noise, congestion and energy security and associated health benefits as a consequence of increased physical activity and personal energy consumption on rates of obesity, diabetes and cardiovascular disease.

Mazzi and Dowlatabadi^48^ have highlighted that climate mitigation policies are a means of reducing fuel consumption and CO_2_ emissions, though caution that these may not necessarily lead to associated health benefits. A switch from petrol to diesel fuelled vehicles may impact on green house gas reduction, but could impact negatively on air pollution and health due to an increase in health-damaging emissions e.g. black carbon emissions from diesel engines.^48^ In addition to the above, Haines et al^29^ highlight the need for policies and incentives that make walking and cycling a more attractive option; these include issues such as legal priority and reallocation of street space and time, making trips more pleasant and attractive.

#### Energy use

Haines^49^ outlines the potential health benefits of renewable energy sources and policies that promote sustainability and use of renewable energy sources, describing how emissions of greenhouse gases can be reduced by increased use of renewable sources, such as wind power in the UK, greater energy efficiency and other measures to promote sustainability. Haines also highlights the considerable health benefits in the short term resulting from policies to reduce the combustion of fossil fuels, particularly those associated with high levels of pollution, such as oil and coal, with estimates from the Working Group on Fossil Fuels and Public Health of some 700,000 lives saved as a result of reductions in greenhouse gas emissions. Haines^49^ refers to a report from the UN Intergovernmental Panel on Climate Change (IPCC) which has reviewed a range of technical options in relation to renewable energy use and its potential for health benefits. Haines et al^29^ consider the importance of policies in promoting access to clean and sustainable energy sources and their potential to both improve public health whilst at the same time mitigating the effects of climate change.^29^ They identify the need for policies that will transform patterns of power generation and energy use in order to address climate change and to improve public health, and to help reduce the vulnerability of poorer populations to the effects of climate change. A number of technology and policy options for the mitigation of greenhouse gas emissions and use of energy in different sectors are considered, for example: energy efficiency and conservation; fuel shift; carbon dioxide capture and storage; nuclear fission; renewable electricity and fuels; forests and agricultural soils. Brayford^42^ outlines the Climate Change Programme devised by the Government to tackle climate change and the recent Energy White Paper, Our Energy Future—Creating a Low Carbon Economy^50^ which sets out the UK’s energy policy which aims to encourage the use of renewable energy and improving energy efficiency in buildings and procurement.

#### Buildings and settlements

Hales et al^30^ emphasises the need for planners of housing and human settlements to consider energy efficiency alongside other criteria for sustainability such as the local impacts of housing design on human health and biodiversity. They use heat waves as an example of the effects and response measures in terms of buildings and health and of the need for land use planning to restrict building on flood plains, coastal areas and heat wave and drought-prone areas. Brayford^42^ refers to environment and building policies/windows such as the NHS Estates’ New Environmental Strategy for the NHS^51^ which aims to address sustainability in an holistic way by ensuring that healthcare buildings are waste reducing and energy efficient, whilst at the same time patient focused. The emphasis is on ensuring a healthcare environment that is not only sustainable but one that also maximises social and health benefits. The need for sustainability policies covering such areas as town planning have been highlighted by Hanlon and McCartney^45^ as essential to address shortfalls in energy supply, rising energy prices and energy vulnerability associated with ‘peak oil’ (see sustainability theme). Haines et al^29^ considers housing and the built environment and indicates that many of the policies that mitigate the effects of climate change will inevitably involve changes that will also serve to enhance the built environment. For example, reducing traffic congestion and pollution and increasing the number of trees in urban environments will make areas more attractive for safe and healthy active transport.

#### Behaviour modification strategies

Coote^46^,^52^ and Mohan et al^44^ describe the good corporate citizen model as a means by which NHS organisations can embrace sustainable development and tackle health inequalities. Griffiths et al^53^ describe the use of doctors and other health professionals as advisors or role models in tackling climate change and for championing associated health benefits. McCartney et al^40^ outlines the need for changes in mindset (including values, attitudes, norms and behaviours) which are required along with clear definitions of the problems faced in terms of economics, society and culture in order to respond to climate change.

### Actions/examples

Table 5 provides a summary of examples of action on climate change and energy vulnerability in relation to health as reported in the literature. This action can be broadly grouped under the following themes: methods that support sustainable development; governance and attitude/behaviour change; gardening and food; energy saving/waste management; transport.

#### Methods that support sustainable development

In considering the potential for the use of *Health Impact Assessment* to test for and promote health and sustainability, Barton and Grant^54^ highlight some of the problems with planning processes for new developments and the lack of emphasis on the interplay between individual health, lifestyle, social networks and environment. They advocate the use of an integrated appraisal approach that embraces economic, social and environmental aspects of sustainable development (the Spectrum Approach). They provide an example of this approach as it has been used in practice and illustrate the stages of the appraisal process, stating that ‘Spectrum is expressly designed to recognize both the integrated agendas of health and sustainable development and the need for an inclusive communicative process’ (p134). Few^55^ uses Health Impact Pathways as an approach to assessing event occurrence (physical risk), social vulnerability and coping capacity, and links this approach with population health and disaster management, stressing the importance of contextual and societal factors that constrain or promote coping efforts. This method focuses on risk reduction at pre-event, event, and post event stages, thus considering both mitigation and adaptation. The Department of Health Health Action Zones initiative provides an opportunity to link sustainability with health, environment and social justice, and to address inequalities in health.^56^ Drawing on the work carried out in Sandwell, in the West Midlands of the UK, Middleton provides an example of regeneration projects, environmentally sustainable buildings, and community food co-operatives; initiatives aimed at promoting local enterprise and building resilience.

#### Governance and attitude/behaviour change

Jeffrey^57^ highlights the World Humanity Action Trust (WHAT) report emphasis on governance ‘in terms of inter-relationships between systems and structures of governance, emphasising the need to take into account all relevant aspects of problems in an integrated way rather than as a series of single-issues’ (p606). This requires organisations to change behaviour and implement action to promote multidisciplinary working. Jeffrey provides an example of an attempt to address water quality through this approach. Tudor et al^58^ found that NHS employees who reported recycling behaviour at home were also more likely to report recycling at work, and that such reports were influenced by underlying attitudes and beliefs about the environment. However, these self-reports of behaviour did not necessarily translate into actual behaviour when measured through observations and waste-bin analysis.^59^ Suggestions for behaviour change at an organisational level included training managers, with an emphasis on cost-savings, increased communication about recycling and environmental issues through staff training and development.^59^

#### Gardening and food

Efforts to reduce food miles and provide healthy local food have been implemented, reducing food miles by two thirds in one NHS Trust.^25^,^60^ Health Action Zone initiatives have promoted food co-operatives and enhanced the local supply of healthy food.^56^ Holland^61^ examined the community garden movement in the UK. ‘Health Issues’ was an important feature of the original purpose of the 96 community gardens surveyed. Connecting purposes were apparent from the data, in particular: education, health, food provision and leisure were linked. Thirty five of the garden projects linked health with skills, suggesting the application of food growing to skills and health promotion.^61^

#### Energy saving/waste management

Efforts to reduce carbon emissions and improve energy efficiency, through, for example, the installation of combined heath and power plants can also bring savings to NHS Trusts;^60^,^62^ wind turbines and solar panels can provide energy and save costs.^25^ Sustainable building programmes can encourage environmentally sound housing, as well as engagement of the local community (for example those with learning difficulties) and provide healthier domestic environments.^56^ More effective management of clinical waste has been achieved by changing from disposable to reusable nappies.^25^

#### Transport

Green travel plans encourage cycling and public transport use,^25^,^60^ with the associated benefits of increasing physical activity.

## Discussion

Interest in the health impacts of climate change has increased significantly in recent years as evidenced by the available literature reviewed here. Interest in the health impacts of peak oil is less evident. What is clear from this systematic review is that much of the literature focuses on the specific health impacts and effects of climate change, the need to take action, and reports of strategies and policies. The literature suggests that we face a future in which the fundamentals of our lives will change and one which poses significant challenges for public health and well being.

Health services in the UK have to some extent already begun to produce and adopt policies and strategies that address some of these risks; waste, transport and energy have all been highlighted in the literature. However, unless these policies are effectively implemented, they will amount to little actual change. Although evidence of concrete actions taken and successful implementation of policy has been demonstrated by this systematic review, the evidence of action was dwarfed by the evidence of well-intended policies and strategies that have yet to be implemented. Furthermore, it is also evident that there is very little in the way of original research in this area, either in relation to the implementation or evaluation of actions.

Although there remains uncertainty over exactly how climate change will affect the UK, it is clear that the environmental problems and effects associated with it will impact most heavily on the poorest and most vulnerable members and regions of society, including older people, children, and low-income families and it is this disproportionate effect on the most disadvantaged in society that is likely to further contribute to health inequalities.^63^ This suggests a fundamental need for action that addresses both sustainability and health inequalities, as many of the steps taken to reduce the effect of climate change will have positive health benefits. For example, efforts to improve the energy efficiency of housing and the local growing of food will have health co-benefits and may go some way to help to reduce the gap between the rich and poor.^18^ In addition, increased use of active transport, such as walking and cycling, along with increased use of public transport as opposed to private cars, will help reduce pollution whilst at the same time improving the health of the population, with improvements in respiratory and cardiovascular health and reduced incidence of obesity and related disorders.^64^

This review is limited by its emphasis on papers published in journals that are indexed on health and biomedical electronic databases. Other databases may have provided a wider literature that could be relevant for or extrapolated to health. Furthermore, other approaches such as searching websites for examples of actions and implementation may prove more successful.^21^ However, actions that have been taken and the policies that have been implemented should be made available for thorough evaluation and discussion within the published literature, in order that others working in healthcare and healthcare education may become better informed and encouraged to take further steps to embed action on climate change and sustainability within their own organisations and working lives.

In the context of the education of healthcare practitioners, neither the UK NHS knowledge and skill framework (KSF) nor the Nursing and Midwifery Council’s Standards of Proficiency for Pre Registration Education^65^ explicitly refer to sustainability and climate change, so it could be argued that at the moment they are still considered marginal concepts for mainstream healthcare education. There is much competition, both ideologically and for space within the healthcare education curricula with current priorities focused on the need to provide trained professionals fit for work to meet the complex needs of a national (and local) NHS in terms of workforce requirements.^66^ Debates around what healthcare workers are *for* must be seen in this context. This is compounded by post registration/qualification education where it is at least arguable that priorities are for ‘training’ to meet local service needs. This may result in the wholesale loss of contracting for courses that do not explicitly address local training needs for service delivery. A course on ‘understanding and analysing global interconnectedness, environment and health’ may seem too ‘ivory tower’ for a health service which must justify its spending. Due to education contracting for specific professional roles there is an underlying drive for educational programmes to meet narrowly defined needs that may not meet future demands.

However, the Department of Health in the UK has clearly set out the context in which NHS Trusts operate and this explicitly includes sustainable development.^67^,^68^ If this context does not help to drive workforce development and education in addressing sustainability and climate change, an opportunity will have been missed.

## Conclusion

There are clearly a large number of papers reporting the potential global health effects of climate change, as well as those reporting policies and strategies in the UK to tackle these effects. However there is an urgent need to identify and report on actual implementation of strategies to mitigate and adapt to these challenges and to publish real examples of actions. The ‘policy—implementation gap’ found in literature relating to the UK is likely to be prevalent in other countries and warrants investigation. Furthermore, any actions that are taken need to be evidence/policy based, and implementations monitored, evaluated and published. Significant funding is required to support evaluation and research in this area, and to assess, for example the impact of initiatives such as the UK NHS Carbon Reduction Strategy.

## Figures and Tables

**Table 1.** Excluded and background papers.

**Authors**	**Year**	**Title**	**Journal**	**Vol, Issue, page**	**Reason for exclusion**
Adshead F and Thorpe A.^69^	2007	The role of the Government in public health: a national perspective	Public Health	121 (11). p. 835–839	Focuses on legislation
Appleby J.^2^	2007	Data briefing. How climate change will affect health	The Health Service Journal	117 (6057). p. 21–21	Impact of climate change on Health (Data briefing) Exclude—data briefing/editorial document
Ahmad B.^70^	2007	Coherent and comprehensive coverage	Impact assessment and project appraisal	25 (1). p. 70–71	Book review
Armstrong R, Waters E, Moore L, Riggs E, Cuervo LG, Lumbiganon P and Hawe P.^71^	2008	Improving the reporting of public health intervention research: advancing TREND and CONSORT	Journal of Public Health	30 (1). p. 103–109	Does not address climate change/sustainability health issues
Bird SR.^72^	2003	African carrier oils: linking aromatherapy, ethnobotany, fair trade, socio-economics and sustainability	Aromatherapy Today	p. 30	Does not address climate change/sustainability health issues
Boykoff MT and Rajan SR.^73^	2007	Signals and noise. Mass-media coverage of climate change in the USA and the UK	EMBO Reports	8 (3). p. 207–211	Review of media reports
Brown RJC, Yardley RE, Muhunthan D, Butterfield DM, Williams M, Woods PT, Brown AS and Goddard SL.^74^	2008	Twenty-five years of nationwide ambient metals measurement in the United Kingdom: concentration levels and trends	Environmental Monitoring and Assessment	142 (1–3). p. 127–140	Does not address climate change/sustainability health issues
Butcher C, McDonald B and Westhorp V.^75^	2003	Healthchecks and sustainable livelihoods: a case study from Kent	Community development journal	38 (3). p. 225–234	A comparison of two approaches to community development: the sustainable livelihoods approach and the healthcheck approach (Original research) Exclude—not health
Carlisle D.^76^	2008	Future NHS. NHS 60. The heat is on	The Health Service Journal	p. 28–31	Personal opinion
Catford J.^77^	2008	Food security, climate change and heath promotion: opening up the streams not just helping out down stream	Health Promotion International	23 (2). p. 105–108	Food security, climate change and health promotion (editorial—therefore exclude)
Chan WM.^78^	2007	Access and Sustainability Advisory Service	Access by Design	p. 14	Does not address climate change/sustainability health issues
Coote A.^14^	2006	What health services could do about climate change	British Medical Journal (Clinical Research Ed.)	332 (7554). p. 1343–1344	Role of health service in tackling climate change (editorial—exclude)
Coote A.^79^	2008	How should health professionals take action against climate change?	British Medical Journal	336 (7647). p. 733–734	Role of health professionals in tackling climate change (editorial—exclude)
Curry A.^80^	2004	Emerging human protozoan infections in the temperate European climate	Journal of Submicroscopic Cytology And Pathology	36 (2). p. 105–119	Focuses on communicable disease control
Darier E and Schule R.^81^	1999	Think globally, act locally’? Climate change and public participation in Manchester and Frankfurt’	Local environment	4 (3). p. 317–330	Excluded from the systematic review but used for background reading/discussion
Davis AM.^82^	2007	Climate change. Why the NHS must think about sustainability	British Journal of Health Care Management	13 (7). p. 264–264	Excluded from the systematic review but used for background reading/discussion
Dobson R.^83^	2008	Obesity and climate change could be tackled together	British Medical Journal (Clinical Research Ed)	336 (7657). p. 1333–1333	Editorial/News item
el Ansari W.^84^	2003	Educational partnerships for public health: do stakeholders perceive similar outcomes?	Journal of Public Health Management and Practice	9 (2). p. 136–156	No reference to climate change
Farmer J, Lauder W, Richards H and Sharkey S.^85^	2003	Dr. John has gone: assessing health professionals’ contribution to remote rural community sustainability in the UK	Social science and medicine	57 (4). p. 673–686	Excluded from the systematic review but used for background reading/discussion
Field J.^86^	2006	Access and Sustainability Advisory Service	Access by Design	p. 25	Does not address climate change/sustainability health issues
Ford S.^87^	2008	Climate change will lead to more kidney stones	Nursing Times	104 (29). p. 8–8	Not UK based
Gibbons J.^6^	2008	Our greatest health menace	World of Irish Nursing and Midwifery	16 (3). p. 47–47	Excluded from the systematic review but used for background reading/discussion
Gill M.^88^	2008	Why should doctors be interested in climate change?	British Medical Journal	336 (7659). p. 1506–1506	Excluded from the systematic review but used for background reading/discussion
Gill M, Godlee F, Horton R and Stott R.^15^	2007	Doctors and climate change	British Medical Journal	335 (7630). p. 1104–1105	Climate change and role of health professionals in tackling effects (Editorial—exclude)
Gould EA, Higgs S, Buckley A and Gritsun TS.^89^	2006	Potential arbovirus emergence and implications for the United Kingdom	Emerging Infectious Diseases	12 (4). p. 549–555	Focuses on communicable disease control
Graham I.^90^	2002	Nursing and developmental sustainability. A personal reflection	Reflections on Nursing Leadership/Sigma Theta Tau International, Honor Society of Nursing	28 (4). p. 27	Excluded from the systematic review but used for background reading/discussion
Griffiths J.^91^	2006	Mini-symposium: health and environmental sustainability. The convergence of public health and sustainable development	Public Health	120 (7). p. 581–584	Health and environmental sustainability (editorial—exclude)
Guillebaud J and Hayes P.^92^	2008	Population growth and climate change: universal access to family planning should be the priority	British Medical Journal	337 (7664). p. 247–248	Excluded from the systematic review but used for background reading/discussion
Harrison D.^1^	2006	Peak oil, climate change, public health and well-being	Journal of The Royal Society of Health	126 (2). p. 62–63	Excluded from the systematic review but used for background reading/discussion
Holyoake D, Wheatley F, Brown J and Chapman K.^93^	2007	Readers panel. Impact on climate change	Nursing Standard	21 (32). p. 24–25	Letter/personal opinion
Howatson G.^94^	2004	Exercise and Hot Spots	Sportex Health	20 p. 28–30.	Exercise specific
Hunt G.^95^	2006	Climate change and health	Nursing Ethics	13 (6). p. 571–572	Excluded from the systematic review but used for background reading/discussion
Johnson EK, Moran D and Vinten AJA.^96^	2008	A framework for valuing the health benefits of improved bathing water quality in the River Irvine catchment	Journal of environmental management	87 (4). p. 633–638	Focuses on communicable disease control
Kashefi E and Mort M.^97^	2004	Grounded citizens’ juries: a tool for health activism?	Health Expectations: An International Journal of Public Participation In Health Care and Health Policy	7 (4). p. 290–302	Focuses on citizens juries
King D.^3^	2007	Climate change challenge laid before public health workforce	Journal of the Royal Society for the Promotion of Health	127 (5). p. 195–195	Excluded from the systematic review but used for background reading/discussion
Konidari P and Mavrakis D.^98^	2007	A multi-criteria evaluation method for climate change mitigation policy instruments	Energy policy	35 (12). p. 6235–6257	Quantitative evaluation methods for climate change mitigation policy instruments (Evaluation research) No reference to health—exclude
Lambert J.^13^	2008	Social care must prepare for the effects of climate change	Community Care	(1704). p. 29–29	Excluded from the systematic review but used for background reading/discussion
Lea R.^99^	2008	The days of cheap oil have gone, but the peak oil theory is far too bleak	Public Health	122 (7). p. 667	Does not address climate change/sustainability health issues
Lewis S and Andrews GJ.^100^	2009	Climate change and health: Priorities for the CAM community	Complementary Therapies In Clinical Practice	15 (1). p. 1–4	Excluded from the systematic review but used for background reading/discussion
Lonsdale J.^101^	2007	Only YOU can save the Earth!	Journal of the Royal Society for the Promotion of Health	127 (5). p. 204–205	Excluded from the systematic review but used for background reading/discussion
Lorenzoni I, Nicholson-Cole S and Whitmarsh L.^102^	2007	Barriers perceived to engaging with climate change among the UK public and their policy implications	Global environmental change	17 (3–4). p. 445–459	Excluded from the systematic review but used for background reading/discussion
Lorenzoni I, Leiserowitz A, De Franca Doria M, Poortinga W and Pidgeon N.^103^	2006	Cross National Comparisons of Image Associations with Global Warming and Climate Change Among Laypeople in the United States of America and Great Britain	Journal of risk Research	9: p. 265–281	Does not address climate change/sustainability health issues
Maryon-Davis A, Gilmore I and Hamilton P.^5^	2007	Climate change and health. We must all act now...	British Medical Journal	335 (7630). p. 1110–1110	Excluded from the systematic review but used for background reading/discussion
Mayor S.^11^	2008	NHS should bring in measures to reduce its carbon footprint, BMA says	British Medical Journal	336 (7647). p. 740	Excluded from the systematic review but used for background reading/discussion
Milner SJ, Bailey C, Deans J and Pettigrew D.^104^	2005	Integrated impact assessment in the UK-use, efficacy and future development	Environmental impact assessment review	25 (1). p. 47–62	Excluded from the systematic review but used for background reading/discussion
Morrison L.^105^	2008	Climate change... what can we do?	British Journal of General Practice	58 (549). p. 290–290	Climate change and the role of GPs Exclude—conference report
Moseley MJ and Owen S.^106^	2008	The future of services in rural England: the drivers of change and a scenario for 2015	Progress in planning	69 (3). p. 93–130	Excluded from the systematic review but used for background reading/discussion
Myr R.^107^	2008	Climate change: Breastfeeding tackles both obesity and climate change	British Medical Journal	336 (7659). p. 1454–1454	Climate change and obesity: the benefits of breastfeeding. Exclude—Letter/personal opinion
Nicoll A and Murray V.^108^	2002	Health protection--a strategy and a national agency	Public Health	116 (3). p. 129–137	Does not address climate change/sustainability health issues
O’Dowd A.^12^	2007	NHS is told it must play its part in tackling climate change	British Medical Journal	334 (7608). p. 1343–1343	Excluded from the systematic review but used for background reading/discussion
Pan H and Kohler J.^109^	2007	Technological change in energy systems: learning curves, logistic curves and input-output coefficients	Ecological economics	63 (4). p. 749–758	Does not address climate change/sustainability health issues
Pidgeon NF, Lorenzoni I and Poortinga W.^110^	2008	Climate change or nuclear power—No thanks! A quantitative study of public perceptions and risk framing in Britain	Global environmental change	18 (1). p. 69–85	Does not address climate change/sustainability health issues
Randolph SE.^111^	2008	Dynamics of tick-borne disease systems: minor role of recent climate change	Revue Scientifique Et Technique (International Office Of Epizootics)	27 (2). p. 367–381	Focuses on communicable disease control
Roberts I.^7^	2008	The economics of tackling climate change	British Medical Journal	336 (7637). p. 165–166	Excluded from the systematic review but used for background reading/discussion
Sim F and Mackie P.^112^	2006	Climate change and health	Lancet	367 (9528). p. 2039–2039	Excluded from the systematic review but used for background reading/discussion
Smith B.^113^	2008	Climate change: Why so many open windows?	British Medical Journal	336 (7659). p. 1454–1454	Letter/personal opinion
Smith C.^114^	2007	Care closer to home. Keeping it real	The Health Service Journal	117 (6065). p. sup.l 9	Does not address climate change/sustainability health issues
Smith S and Swierzbinski J.^115^	2007	Assessing the performance of the UK emissions trading scheme	Environmental and resource economics	37 (1). p. 131–158	Does not address climate change/sustainability health issues
Smith PF and Twisselmann B.^116^	2006	‘Climate change... Stott R. Healthy response to climate change (with commentary by M Hillman, L Eaton)	British Medical Journal	332 (7556). p. 1509–1509	Excluded from the systematic review but used for background reading/discussion
Stevenson WT.^117^	2006	Doctors leading climate change is self delusion	British Medical Journal	333 (7578). p. 1124–1124	Letter/personal opinion
Stanton J.^118^	2008	MiniSymposium. Sustainability: public health’s role in the 21st century	Public Health	122 (7). p. 645–646	Excluded from the systematic review but used for background reading/discussion
Stott R.^38^	2007	Actions to mitigate climate change—a view from 2057	British Medical Journal	335(7633): 1318–1319.	Personal opinion
Stott R.^18^	2006	Contraction and convergence. Healthy response to climate change	British Medical Journal	332 (7554). p. 1385–1387	Excluded from the systematic review but used for background reading/discussion
Stott R and Godlee F.^8^	2006	What should we do about climate change? Health professionals need to act now, collectively and individually	British Medical Journal	333 (7576). p. 983–984	Excluded from the systematic review but used for background reading/discussion
Tudor T, Barr S and Gilg A.^20^	2008	A novel conceptual framework for examining environmental behaviour in large organizations: a case study of the Cornwall National Health Service (NHS) in the United Kingdom	Environment and behavior	40 (3). p. 426–450	Excluded from the systematic review but used for background reading/discussion
Twisselmann B.^22^	2006	Climate change: Summary of responses	British Medical Journal	332 (7556). p. 1509	Excluded from the systematic review but used for background reading/discussion
Watson R.^119^	2007	UN conference on climate change will test countries’ commitment to public health	British Medical Journal	335 (7630). p. 1116–1117	Brief conference report
While A.^120^	2006	Climate change should matter to nurses	British Journal of Community Nursing	11 (10). p. 454–454	Excluded from the systematic review but used for background reading/discussion
Wilkinson P.^41^	2008	Peak oil: threat, opportunity or phantom?	Public Health	122 (7). p. 664	Excluded from the systematic review but used for background reading/discussion
Williams N.^121^	2006	Costing climate change	Current Biology: CB	16 (23). p. R971–972	Editorial/News item
Wilson N and Wallace C.^122^	2006	Climate change control and injury prevention: more win-win solutions	Injury Prevention	12 (2). p. 135–135	Climate change control and injury prevention solutions: price incentives via carbon charges for demand reduction, increased energy efficiency (Letter in response to) Exclude—Letter/personal opinion
Zarocostas J.^123^	2008	WHO chief calls for united front in face of three crises: food, climate change, and pandemic influenza	British Medical Journal	336 (7654). p. 1155–1155	Editorial/News item
Not known	2007	Climate change action must not forget vulnerable households^124^	Working with Older People: Community Care Policy and Practice	11 (3). p. 7–7	Excluded from the systematic review but used for background reading/discussion
Not known	2007	NHS has a major role to play in tackling climate change^125^	Nursing in Practice: The Journal for Today’s Primary Care Nurse	(36). p. 7–7	Excluded from the systematic review but used for background reading/discussion
Not known	2007	Public health must be part of climate change ‘jigsaw^126^	Journal of the Royal Society for the Promotion of Health	127 (6). p. 244–244	Excluded from the systematic review but used for background reading/discussion
Not known	2008	Adapting to climate change^127^	Lancet	371 (9613). p. 624–624	Excluded from the systematic review but used for background reading/discussion
Not known	2008	Trusts urged to recruit locally to save the planet^128^	Nursing Standard	22 (32). p. 5–5	Excluded from the systematic review but used for background reading/discussion

**Table 2.** Characteristics of included articles.

**Name**	**Category**	**Focus**
Campbell^25^	Problems/Impacts/Effects	Climate change and role of health professionals in relation to adaptation and mitigation Health impacts of climate change: 3 kinds of health impacts were identified (direct caused by weather extremes e.g. Loss of crops; the health consequences of environmental change and ecological disruption e.g. Rise in sea levels and spread of malaria; diverse health consequences due to population displacement e.g. Mental health consequences) Global and direct impact of climate change: Impacts of climate change on health in UK of two types: global impacts e.g. Food insecurity due to crop failures; increase in armed conflict over water, land and food with population displacement Initiatives for tackling climate change: Examples of successful NHS initiatives (e.g. Addenbrookes Hospital, Cambridge and sustainable transport policy; Antrim Area Hospital, installation of wind turbines to reduce fuel costs; Bronllys Hospital, Wales installation of solar panels; Noble’s Hospital, Isle of Man use of reusable nappies to reduce clinical waste; Royal Cornwall Hospital Trust in partnership with the Soil Association to increase amount of locally sourced hospital food Co-benefits: positive health benefits associated with steps to mitigate climate change outlined e.g. Improvement in air quality with reduction in car use would also lead to better respiratory health and fewer premature deaths; increase in physical activity would also reduce incidence of obesity and obesity related illnesses
Diffey^32^	Problems/Impacts/Effects	Climate change, ozone depletion and impact on UV exposure of human skin Consequence of climate change and impact of behaviour change on UV exposure: ‘….climate change could have a greater impact on future skin cancer incidence in northern Europe than ozone depletion due to changes in behaviour encouraging more time in the sun than increases in ambient UV’
Donaldson^34^	Problems/Impacts/Effects	Climate change and respiratory syncytial virus epidemics (original research) Health benefit of climate change in UK: this study reports ‘…these findings imply a health benefit of global warming in England and Wales associated with a reduction in the duration of RSV season and its consequent impact on the health service’
Goodwin^31^	Problems/Impacts/Effects	Winter mortality in the elderly and effect of climate change (Review) Integrated health and social policy for tackling winter mortality in older people and role of anticipatory community health care; health behaviours for reducing risk e.g. ‘….specifically, older people need to be aware of habitual behaviours and attitudes that place them at risk…the solution to the excess winter death problem will require concerted effort to improve not only the indoor warmth of all older people, but to ensure that so-called ‘high risk’ behaviour is mitigated’
Haines^49^	Problems/Impacts/Effects	Sustainability, energy consumption and economic growth and effects on public health (Discussion paper) They illustrate with an example of use of solar energy combined with energy efficiency measures (Rocky Mountain Institute Headquarters in Colorado http://www.rmi.org/sitepages/pid229.php)
Haines et al^26^	Problems/Impacts/Effects Also Policy/Strategy	Climate change, impacts, vulnerability and public health: adaptation and mitigation strategies (Mini-symposium)
Hales et al^30^	Problems/Impacts/Effects Also Policy/Strategy	Climate change and effects on housing, human settlements and health: adaptation and mitigation of effects (Review)
Hunter^27^	Problems/Impacts/Effects	Climate change and impact on health from waterborne and vector-borne infections (Review)
Mazzi and Dowlatabadi^48^	Problems/Impacts/Effects	Effects of climate mitigation policies in UK in relation to air quality
Montgomery^28^	Problems/Impacts/Effects	Climate change and health implications (Review)
Sinclair^33^	Problems/Impacts/Effects	Depletion of ozone layer and effects on health (Review)
**Name**	**Category**	**Focus**
Brayford^42^	Strategy/Policy	Sustainability in the NHS: co-benefits and actions around 5 key themes of energy, water, waste, transport and procurement (Discussion paper)
Caraher and Coveney^36^	Policy/strategy	Public health nutrition and food policy (review)
Coote A^52^	Policy/strategy	Sustainability and health
Coote A^46^	Policy/strategy	Labour’s health policy
Fussel HM^129^	Policy/strategy	Climate change adaptation assessment for reducing associated health risks (review)
Griffiths et al^90^	Policy/Strategy	Role of doctors in tackling climate change
Haines Smith Anderson et al^29^	Policy/strategy	Policies for enhancing access to clean energy, improving health, advancing development and mitigating climate change
Hanlon and McCartney^45^	Policy/strategy	Peak oil and resultant challenges to public health; potential for economic planning and sustainable development as mitigation (Mini-symposium)
Mohan et al^44^	Policy/strategy	Waste management in the UK and the role of public health (review)
Roberts and Arnold^47^	Policy/strategy	Climate change policy and injury control (Policy forum) Transport policy—transition to low-carbon, low-energy transport system Co-benefits of climate change policies: e.g.Reductions in volume and speed of traffic, particularly in cities, could mitigate climate impacts, reduce injury rates, and improve air quality. Increasing levels of active transport will have important consequences for physical activity and would impact on rates of obesity, diabetes and cardiovascular disease. Other potential co-benefits include reduced noise and congestion and energy security
Roberts and Hillman^37^	Policy/strategy	Climate change and implications for policy on injury control and health promotion (Special feature article)
Stott^18^	Policy/strategy	Contraction and convergence—policies for tackling climate change (Analysis and comment)
Stott^38^	Policy/strategy	Climate change, poverty and war/security implications. Contraction and convergence strategy (Essay)
Wilkinson^39^	Policy/strategy	Climate change and health and the case for sustainable development (adaptation and mitigation) (Discussion paper)
Barton and Grant^54^	Actions/Examples	Sustainability and health: a review of theory and practice around environmental impact analysis and health impact assessment (Review)
Campbell^25^	Actions/Examples	Reports successful NHS initiatives
Fairchild and Morgan^35^	Actions/Examples	The Cardiff Food Strategy case study: the development of a sustainable food and health strategy (Original research)
Few^55^	Actions/Examples	Climate change and health impact: a consideration of vulnerability, response and adaptation from social research
Holland^61^	Actions/Examples	Community development programmes and sustainable development: an analysis of the community garden movement (Original research)
Jeffery^57^	Actions/Examples	Public health and sustainable development: examples of water quality regulation (Mini-symposium)
McCartney et al^40^	Actions/Examples	Climate change, health, equality and sustainability: challenges to public health and potential actions (Mini-symposium)
Middleton^56^	Actions/Examples	Link between health, environmental and economic sustainability: examples of policy and programme development for public health improvement (Review)
Moore^60^	Actions/Examples	Climate change and the NHS: examples of mitigation approaches by individual Trusts (Brief report)
Potter^62^	Actions/Examples	Impacts of climate change on health: role of NHS in mitigating effects (Feature article)
Smith and Haigler^130^	Actions/Examples	Co-benefits of climate mitigation and health protection in energy systems: examples of scoping methods for assessment (Review)
Tudor, Barr and Gilg^20^	Actions/Examples	Strategies for improving recycling behaviour and promoting sustainability within the Cornwall NHS (Original research)
Tudor, Barr and Gilg^58^	Actions/Examples	Sustainable waste management behaviour: factors influencing ‘home’ and ‘work’ behaviours (Original research)
**Name**	**Category**	**Focus**
Stott^38^	Co-benefits	Climate change: contraction and convergence (Discussion article)

**Table 3.** Impacts/Effects: Analysis of themes.

**Theme**	**Descriptive terms (reference)**
**Water-borne disease**	Increase in spread of vector mosquitoes with a resultant increase in incidence of water-borne diseases such as malaria^2^,^25^,^26^ though this is unlikely in UK.^28^ Increased incidence of cholera associated with rising temperatures.^27^
**Vector-borne disease**	Increases in insect-borne disease from flies and ticks due to changes in land use and leisure activities as opposed to climate change.^25^ Changes in the incidence, distribution and transmission of tick-borne encephalitis.^26^
**Food-borne disease**	Increased incidence of food poisoning as indirect result of climate change and flooding;^25^,^30^ dengue and diarrhoeal disease, resulting in an increased risk of food contamination.^2^,^26^–^28^
**Incidence of temperature related deaths**	Increase in mortality and thermal stress in hot weather due to increased frequency of heat waves associated with climate change^26^,^28^,^29^ though this is most likely to affect vulnerable individuals i.e. the elderly. Hales et al 2007^30^ health impacts of heat waves. Reduction in cold-related deaths associated with climate change and rising temperatures e.g^26^,^28^ though this is likely to be minimal.^31^
**Skin cancer and cataracts**	Increase in incidence of skin cancer and cataracts as result of increased UV exposure and associated ozone depletion.^32^,^33^
**Increase in starvation/malnutrition**	Loss of staple crops and impact on nutrition and health and development.^25^,^26^ Impact of climate change and rising sea levels in coastal areas on local agriculture infrastructure, goods and transport.^25^,^26^ Increase in malnutrition and consequent disorders with implications for child growth and development.^28^ Increase in migration and armed conflict over land.^25^
**Increase in respiratory disease and deaths due to pollution/particulate matter**	Increase in cardio-respiratory diseases associated with an increase in ground level ozone and pollution. An increase in particulate matter associated with an increase in diesel fuel consumption in UK.^26^,^28^ Reduction in respiratory syncytial virus (RSV) season with resultant health benefit.^34^
**Injury and death due to flooding/storms**	Increased risk of drowning and other injuries due to flooding/storms.^26^ Contamination due to mobilisation of dangerous chemicals and increased risk of diarrhoeal and respiratory diseases as direct result of flooding.^26^
**Psychological effects**	Effect of flooding, drought, environmental degradation and population displacement on the mental and psychological of populations.^26^,^30^ For example, the effect of flooding, drought and environmental degradation associated with climate change and resultant population displacement and the creation of environmental refugees, creating psychological and mental health problems for the displaced populations.^25^,^26^

**Table 4.** Policy/Strategy: Analysis of themes.

**Theme**	**Descriptive terms (reference)**
**Food and Health**	A comprehensive food policy for delivery of food provision and supply for Cardiff.^35^ Health impact of poor food policy decisions.^36^ Sustainable land-use and food security policies are considered by Haines et al.^27^
**Contraction and Convergence**	Contraction and convergence proposed as the most practical and equitable strategy for reducing CO_2_ emissions and tackling climate change^18^,^37^–^40^
**Water and Waste Management**	Applying Government strategies for waste management and water conservation.^42^ NHS estates working to survey/benchmark for performance monitoring ‘watermark’ initiative.^42^ Involvement of public health professionals in regulation of waste management as part of the Integrated Pollution Prevention and Control (IPPC) process.^44^
**Sustainability**	NHS Estates New Environmental Strategy for the NHS which encompasses buildings, energy, waste, water, transport and procurement.^42^ Sustainability policies covering such areas as energy, transport, town planning and structure of economy.^45^ Policies to promote access to clean and sustainable energy sources.^27^ UK SHA strategies and policies for sustainability and tackling climate change and energy vulnerability.^21^ NHS policies that promote sustainable development to include all aspects of its business.^46^
**Transport**	Transport policies aimed at limiting environmental impact, reduce congestion and improve health.^27^,^42^ Climate mitigation policies as a means of reducing fuel consumption and CO_2_ emissions.^48^ Associated health benefits of transition to a low-carbon, low-energy transport system.^47^ Policies that support active transport options such as walking and cycling.^27^
**Behaviour modification strategies**	Good corporate citizen model.^44^,^46^,^52^ Use of doctors and other health professionals as advisors or role models.^90^ Behavioural change strategies.^40^
**Energy use**	Haines^49^ outlines policies in relation to sustainability, energy use and renewable energy. Policies to promote access to clean and sustainable energy sources.^27^ UK energy policy and plans to reduce pollution and effects of climate change and to increase use of renewable energy sources, such as wind and wave power.^42^
**Buildings and settlements**	Environment and building policies and strategies.^42^ Sustainability policies covering such areas as town planning.^45^ Hales et al.^30^ uses heatwaves as an example of the effects and response measures in terms of buildings. Haines et al.^27^ housing and built environment policies and their associated co-benefits.
**Public health adaptation measures**	Haines et al.^26^ p593. Hales et al.^30^ presents adaptation and mitigation strategies for specific climate change health issues (p297–8).
**UK Government Initiatives**	Brayford^42^ and Campbell^25^ list a number of UK government climate change initiatives: Securing the Future^25^ Draft Climate Change Bill^25^ NHS Environmental Assessment Tool^25^ ‘A Better Quality of Life’ Department of Environment.^42^ ‘New Environmental Strategy for the NHS’ NHS Estates 2000.^42^ ‘The Climate Change Programme’.^42^ ‘Sustainable Development in the NHS’.^42^ ‘NHS Environmental Assessment Tool (NEAT)’^42^ ‘Our Energy Future—Creating a Low Carbon Economy’ White Paper 2003 42 ‘Carbon Trust’ Action Energy Programme.^42^ Directing the Flow—Priorities for Future Water Policy—DEFRA 2002.^42^ ‘Waste Not, Want Not’—A Strategy for Tackling Waste Products in England—2002.^42^ ‘Environmental Protection Act and’ Loss of Crown Immunity for NHS.^42^ ‘Strategy for Total Waste Management’.^42^ ‘New Deal for Transport: Better for Everyone’.^42^ ‘The Transport Strategy’.^42^ ‘Building a Better Quality of Life: A Strategy for More Sustainable Construction’ (DEFRA) (Brayford). ‘Achieving Sustainability in Construction Procurement—Sustainability Action Plan.^42^ ‘Construction Best Practice Programme’ (CBPP).^42^ Measurement for Innovation (M4I)^42^ Green Public Private Partnerships—A Guidance Note on How to Include Environmental Considerations within PPPS and PFI Projects.^42^

**Table 5.** Examples of action: Analysis of themes.

**Theme**	**Descriptive terms (reference)**
**Methods that support sustainable development**	Health Impact Assessment as a method for promoting health and sustainability. Use of sustainability appraisal approach—‘Spectrum’, designed to integrated health and sustainability agendas with participatory process. Outline provided of six-stage appraisal process.^54^ Health Impact Pathways as a method of assessing the potential impacts and vulnerabilities of climate change hazards at different points in time with examples of response mechanisms.^55^ Government initiatives such as Health Action Zones and Healthy Cities programmes provide opportunities for addressing sustainable development and building resilience.^56^
**Governance and attitude/behaviour change**	Example of multi-disciplinary approach to water treatment and water quality based on recommendations from the World Humanity Action Trust.^57^ Links between attitudes and perceptions of recycling behaviour at home and at work,^58^ do not necessarily translate into recycling behaviour.^59^ Strategies are suggested for improving behaviour.^59^
**Gardening and Food**	Sourcing food locally to reduce food miles.^25^,^60^ Food co-operatives and community agriculture, partnerships with farmers to promote local growing, improving supply of fruit to schools.^56^ Community gardens could act as a model for the implementation of social, economic and environmental policies at local level.^61^
**Energy Saving/Waste Management**	Affordable warmth programme, sustainable buildings.^56^ Energy saving equipment for heating and ventilation.^60^ Combined heat and power plant and energy efficiency measures.^62^ Wind turbines and solar panels installed.^25^ Reusable nappies reduce clinical waste.^25^
**Transport**	Green travel plans.^25^,^60^
